# Implications of Antibiotic Use during the COVID-19 Pandemic: The Example of Associated Antimicrobial Resistance in Latin America

**DOI:** 10.3390/antibiotics10030328

**Published:** 2021-03-20

**Authors:** Carlos Álvarez-Moreno, Sandra Valderrama-Beltrán, Alfonso J. Rodriguez-Morales

**Affiliations:** 1 Department Facultad de Medicina, Universidad Nacional de Colombia, Bogota 111176, Colombia; 2 Clínica Colsanitas Grupo Keralty, Clínica Universitaria Colombia, Bogota 111176, Colombia; 3 Hospital Universitario San Ignacio, Bogotá 110231, Colombia; sandra.valderama@gmail.com; 4 Departamento de Microbiología, Pontificia Universidad Javeriana, Bogotá 110231, Colombia; 5 Grupo de Investigación Biomedicina, Faculty of Medicine, Fundación Universitaria Autónoma de las Américas, Pereira, Risaralda 660003, Colombia; 6 Latin American Network of COVID-19 Research (LANCOVID), Pereira, Risaralda 660003, Colombia

Antimicrobials are essential for infection management. The development of antimicrobial resistance (AMR) is a growing public health problem. Globally, by the year 2050, an estimated 10 million annual deaths will be attributable to AMR [1,2]. Multi-drug resistant microorganisms have spread throughout the world, and Latin America is no exception. The reported rates of resistance to carbapenem in 2017 on average were 90%, 50%, and 10% for *Acinetobacter baumannii*, *Pseudomonas aeruginosa*, and *Klebsiella pneumoniae*, respectively, which are associated with poor outcomes, while the reported prevalence of resistance in methicillin-resistant *Staphylococcus aureus* bacteremias was 44.7% [3,4,5].

On the other hand, Latin America has faced critical moments during the COVID-19 pandemic, and is considered one of the world’s epicentres, with overwhelmed health systems. The current increase in Brazil, now with more than 11.6 million cases, making it the country with second highest number of cases after the United States of America, is of utmost concern. It has been necessary to establish intensive care unit (ICU) infrastructures in record time and train human resources, often without the best quality standards. Additionally, many of the infection prevention measures in hospitals for non-COVID-19 patients may be decreased or limited due to a lack of resources or prioritization of these for the care of COVID-19 patients. In addition, the programs for the rational use of antimicrobials and infection control, fundamental pillars in AMR control, are still limited in the region. During the COVID-19 pandemic, comprising more than 120.6 million cases (16 March 2021), these programs face the challenge of maintaining surveillance and education actions, despite having been rapidly overwhelmed.

Another challenge in COVID-19 is the management of antimicrobials in both in- and outpatients. Antimicrobials, such as azithromycin, or the antiparasitic drugs chloroquine, hydroxychloroquine, and ivermectin, which are not effective in the management of this infection, and strategies such as “catracho” (a Spanish term for a person from Honduras, used as an acronym for colchicine, anti-inflammatories, tocilizumab, ivermectin, anticoagulants, and hydroxychloroquine) or “maiz” (in Spanish, “corn”, used as an acronym for Microdacyn^®^, azithromycin, ivermectin, and zinc), are being routinely indicated in several countries in the region, promoted by health care professionals or even by governments and the media, without any supporting scientific evidence [6]. On the other hand, in several countries in the region, direct or online sales of antimicrobials are allowed without a prescription, thus facilitating access to these drugs.

Recently, Vaughn et al. noted that the probability of bacterial coinfection in patients hospitalized with COVID-19 was only 3.5% at the time of presentation. Despite this, the reported use of antibiotics in hospitalized patients with COVID-19 was from 27% to 84% [7]. This finding was corroborated in a recent meta-analysis: the overall proportion of COVID-19 patients with bacterial infection was 6.9%, but 71.9% received antibiotics [8]. Antibiotic consumption is higher in low- and middle-income countries. A higher frequency of healthcare-associated infections caused by multi-drug-resistant germs has also been found in these countries [9,10]. Considering the confluence of this pandemic with the emergence of AMR, it is essential to draw attention to the need not to minimize the impact of antimicrobial overuse. Currently, the great challenge is to build or strengthen both national and institutional infection control and rational antimicrobial use programs that promote and reinforce the necessary measures to contain both public health threats [11]. COVID-19 still is a huge challenge for healthcare systems and infectious disease physicians [12]. There are multiple implications of antibiotics used during the COVID-19 pandemic, as shown in this introductory Editorial, for example in the region of Latin America. In the Special Issue “Implications of Antibiotic use during the COVID-19 Pandemic: Present and Future”, it is expected that multiple articles will address this problem and suggest solutions.

Finally, scientific associations, such as the International Society for Antimicrobial Chemotherapy (ISAC), the Pan-American Infectious Diseases Association (API), or in Colombia, the Association of Infectious Diseases (ACIN), should lead great efforts to deal with this emerging problem during the midst of the COVID-19 pandemic, which threatens to increase the already established problem of the growing AMR in the region.
